# Frailty and Socioeconomic Development in the European Region—Associations with Mortality in Middle-Aged and Older Adults

**DOI:** 10.3390/ijerph23030307

**Published:** 2026-02-28

**Authors:** Rónán O’Caoimh, Aoife Wall, Mark R. O’Donovan

**Affiliations:** 1 Health Research Board Clinical Research Facility, University College Cork, Mercy University Hospital, T12 WE28 Cork City, Ireland; markodonovan@ucc.ie; 2 Department of Geriatric Medicine, Mercy University Hospital, T12 WE28 Cork City, Ireland; aoifek.wall@hse.ie

**Keywords:** frailty, social determinants of health, aging, mortality, health status disparities, Europe

## Abstract

**Highlights:**

**Public health relevance—How does this work relate to a public health issue?**

- Population ageing in Europe is accompanied by cross-national differences in frailty prevalence, reflecting broader social and demographic inequalities.
- This study examines how country-level sociodemographic development (Sociodemographic Index) relates to frailty and mortality in adults aged 50+ across multiple European countries.

**Public health significance—Why is this work of significance to public health?**

- Lower sociodemographic development is associated with substantially higher frailty prevalence, independent of frailty definition, highlighting persistent structural health inequalities within Europe.
- Adjustment for frailty attenuates the association between sociodemographic development and mortality, suggesting frailty may account for an important share of survival differences.

**Public health implications—What are the key implications or messages for practitioners, policy makers and/or researchers in public health?**

- Frailty prevention and early identification may represent actionable targets to mitigate the health consequences of social disadvantage in ageing populations.
- Cross-national monitoring of frailty using harmonised measures can support equity-oriented public health strategies and guide resource allocation in ageing societies.

**Abstract:**

The Sociodemographic Index (SDI) captures a country’s or region’s relative socioeconomic development and has been linked to age-related disease burden and life expectancy. Frailty is a multidimensional geriatric syndrome associated with adverse health outcomes and mortality. This study examined the relationship between country-level SDI, frailty prevalence, and mortality across Europe. We conducted a secondary analysis of community-dwelling adults aged 50 years and older from 12 countries participating in the Survey of Health, Ageing and Retirement in Europe (SHARE). Frailty status and SDI were assessed at Wave 2 (2007), with mortality follow-up at Wave 4 (2011). Countries were categorised into lower- and higher-SDI groups using the median as a cut-off. Frailty was measured using a 70-item frailty index (FI ≥ 0.25) and a modified Fried frailty phenotype (FP ≥ 3 criteria). Frailty prevalence varied substantially by country and assessment method, ranging from 7 to 40% using the FI and 4–21% using the FP. Prevalence was lowest in Switzerland and highest in Poland and was strongly correlated with national SDI scores (r ≥ 0.8). After adjustment for age and sex, lower SDI was independently associated with higher odds of frailty using both frailty measures. Although mortality was lower in higher-SDI countries, this association was not statistically significant after adjusting for age, sex, and frailty. Lower social development was strongly associated with frailty prevalence but did not independently predict mortality, highlighting frailty as a potential pathway linking social context to later-life health outcomes in Europe.

## 1. Introduction

Population ageing is accelerating across Europe and around the world, with substantial heterogeneity in health outcomes between countries [1]. Differences in social and economic development are increasingly recognised as key determinants of population health, influencing life expectancy, disability, and the burden of age-related disease. The Sociodemographic Index (SDI), a composite measure of relative socioeconomic development at the country or regional level incorporating income per capita, educational attainment, and fertility rates, has been widely used to capture relative levels of social development and to contextualise global and regional health disparities. Scored from 0 (lowest) to 1 (highest), it is measured as a composite average of income per capita, mean education in those aged <25 years and total fertility rated in women aged <25 years in a country [2]. Lower SDI scores have been associated with increased mortality, higher disability-adjusted life years, and a greater burden of non-communicable diseases, especially amongst older adults [3]. Comparing the highest to lowest SDI quintile age-related burden of disease was 93% higher (137.8 disability-adjusted life years [DALYs] to 265.9 DALYs) and non-age-related burden of disease was 78% higher (149.3 to 265.9 DALYs) globally [4]. Data from the Global Burden of Diseases (GBD) study has shown that low-SDI countries and territories have greater life disparity and more healthy life disparity, which are important summary measures of overall population health reflecting length and quality of life respectively [5].

The relationship between frailty and socioeconomic status is recognised at the individual level with several studies showing that older adults with frailty have a lower socioeconomic position in society [6,7]. However, less is known about how SDI relates specifically to frailty, a multidimensional age-associated syndrome characterised by increased vulnerability to stressors and a heightened risk of adverse outcomes [8], including hospitalisation, institutionalisation, disability, and death [9]. Frailty represents a critical intermediary between social conditions and late-life health outcomes and may help explain cross-national differences in ageing trajectories [10]. It has a high prevalence in Europe [11] and globally [12], which varies by definition [12]. In Europe, the Survey on Health, Ageing and Retirement in Europe (SHARE), a longitudinal cohort study of those aged ≥50 years including >150,000 participants over nine different waves and across 28 countries, includes data on frailty, which varies from as low as 7% in Switzerland to as high as 41% in Poland using different definitions [13]. An analysis of 15 countries included in the first wave of the SHARE found that a mean frailty index score was negatively correlated with both gross domestic product (r = −0.79; *p* < 0.01) and health expenditure (r = −0.63; *p* < 0.05) in countries [14]. Frailty is also moderately associated with social vulnerability [15].

Frailty can be operationalized using different conceptual models. This analysis will focus on the two most widely available models of frailty, the frailty phenotype (FP) and the deficit accumulation frailty index (FI) [16]. The FP measures frailty as a collection of signs and symptoms usually including at least three of the following: unintentional weight loss, self-reported exhaustion, weakness (grip strength), slow walking speed, and low physical activity [17]. The FI considers frailty as a risk state defined by an accumulation of a large number of non-specific health deficits (such as signs, symptoms, diseases, mental and cognitive issues) [18], and is usually scored as the proportion of deficits out of a list of at least 30 items, with scores ≥ 0.25 indicating frailty [19]. Both approaches are clinically distinct yet complementary definitions of frailty [20]; they often yield different prevalence estimates [12] and may vary in their sensitivity to social and contextual factors [20].

Although previous European studies have demonstrated substantial between-country variation in frailty prevalence [13], few have explicitly examined whether these differences align with country-level SDI scores. Furthermore, it remains unclear whether SDI independently predicts mortality once individual frailty status is taken into account. Understanding these relationships is important for informing public health strategies aimed at reducing health inequalities in ageing populations. Hence, the aim of this study was to examine the association between country-level SDI, frailty prevalence, and mortality in a large, harmonized European cohort, the SHARE. Specifically, we assessed whether SDI was associated with frailty prevalence using two established frailty measures and whether SDI independently predicted mortality after accounting for age, sex, and frailty in the SHARE.

## 2. Materials and Methods

### 2.1. Design

This is a secondary analysis of adults aged ≥50 years from 12 countries in the SHARE [21]. The SHARE includes data from comprehensive questionnaires detailing health, social care, finances, education, and early life. Frailty status and baseline covariates were assessed at SHARE Wave 2 (2007) and survival follow-up (mortality data) at Wave 4 (2011). Mortality in SHARE was ascertained through follow-up contact with consenting relatives, friends, or neighbours, as linkage to census or registry data was unavailable.

### 2.2. Sample and Data Collection

Twelve countries were included from SHARE Wave 2 in 2007 (Austria, Belgium, Czech Republic, Denmark, France, Germany, Italy, Netherlands, Poland, Spain, Sweden and Switzerland). Cross-sectional sampling weights were applied. SDI scores were added for 2006 and 2007 from the GBD 2023. SDI quintiles were provided and all countries were in the “High SDI” group. SHARE participants were included if their country was included in both Waves 2 and 4 of the SHARE, if they were aged ≥50 years and were community-dwelling. They were excluded if they were resident in a nursing home or had missing data (relating to the FI, mortality, or sampling weights). SHARE data were collected using computer-assisted interviews administered by trained staff, with proxy respondents such as relatives, friends, or neighbours included when required [21].

### 2.3. Variables

The SDI is a composite, country-level indicator combining income per capita, educational attainment, and fertility rates among younger women. The SDI provides a relative measure of a country’s position along the social and demographic development spectrum, allowing meaningful cross-national comparisons. Specifically, it is a composite average of income per capita, mean education in those aged <25 years and total fertility rated in women aged <25 years [2]. SDI scores were dichotomized into lower and higher using the median scores as cut-offs. Frailty was measured using a 70-item FI (score ≥ 0.25) and a modified Fried FP (≥3/5 positive criteria). The 70-item FI has been previously described for SHARE Waves 1 and 2 [22]. The FI comprised biological deficits (*n* = 29, 41% of items), including 16 comorbidities and 13 signs or symptoms; cognitive deficits (*n* = 4, 6%); mental wellbeing deficits (*n* = 11, 14%); functional deficits in activities of daily living (*n* = 25, 36%); and two additional items capturing self-rated health and hospitalisation in the previous year (3%). Consistent with established FI methodology [19], low physical activity, reflecting its role as a health-related deficit contributing to physiological vulnerability, was included. Employing both measures allows assessment of whether observed associations with SDI are consistent across differing conceptualisations of frailty.

Additional variables collected included educational level (based on the International Standard Classification of Education Scale, ranging from 0, low, to 6, high), employment status, body mass index (BMI) based on self-reported height and weight, financial insecurity, and self-rated health (SRH), a common measure of health recoded from a five-category scale into a binary variable, with good or very good health coded as 0 and poorer health as 1 [23]. Further variables included the number of hospitalisations in a year, the presence or absence of a cognitive deficit, a long-term health problem, any disability in activities of daily living (ADL) and whether daily activities were limited by health (assessed using the Global Activity Limitation Indicator or GALI, which asks whether respondents have been limited by health problems in usual activities for at least six months [24]), all recorded as binary responses to the questionnaire. Finally, quality of life (QoL) was based on the Control, Autonomy, Self-realization, and Pleasure (CASP) CASP-12 score, a 12-item questionnaire scored from 12 to 48, where higher scores indicate better QoL, reflecting a person’s sense of wellbeing across these four key domains [25]. It is a shorter, modified version of the original 19-item CASP scale.

### 2.4. Statistical Analysis

All analyses were conducted using R version 4.4.2. and the “survey” package was used to apply calibrated cross-sectional survey weights (provided by SHARE) and sampling design adjustments to improve population representativeness. Country-level correlations between SDI scores, frailty prevalence, and mortality rates were examined using Pearson’s correlation coefficients and visualized graphically. QoL, measured using the CASP-12 scale, was analysed descriptively and compared across SDI categories using weighted means and appropriate inferential statistics. CASP-12 was not included as an outcome in regression models but was used to contextualise differences in wellbeing across social development strata.

Descriptive comparisons were performed across SDI categories, with univariate differences assessed using complex-sample General Linear Models for continuous variables and complex-sample Pearson’s chi-square tests for categorical variables. Holm-adjusted *p*-values were applied to account for multiple comparisons. Associations between SDI and mortality were examined using complex-sample logistic regression, estimating odds ratios (ORs) and 95% confidence intervals (CIs). Models were adjusted for age and biological sex, and additional models accounted for frailty status. Predicted probabilities were derived from the models including B-Splines to allow non-linear associations with SDI score. Survival analysis was performed using Kaplan–Meier curves for visualisation and Cox proportional hazards modelling to examine the adjusted Hazard Ratio (HR). Statistical significance was set at *p* < 0.05.

## 3. Results

**Figure 1:**
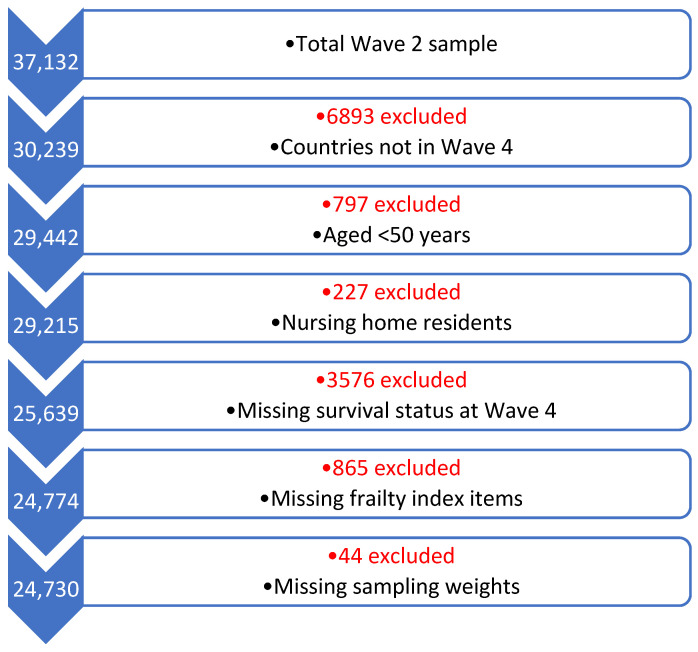
Selection of participants in Survey of Health, Ageing and Retirement in Europe Waves 2 to 4.

**Figure 2:**
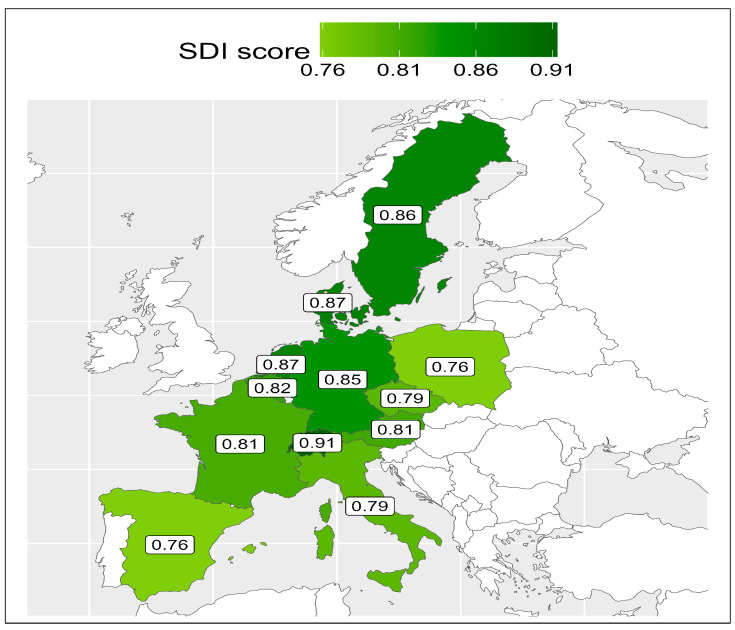
Mean Sociodemographic Index (SDI) scores for each country included in the Survey of Health, Ageing and Retirement in Europe Waves 2 to 4 (countries scoring 0.82 or less were below the median).

At SHARE Wave 2, 37,132 participants were initially available. After applying inclusion and exclusion criteria, 24,730 people from 12 countries were available and included in this analysis. The selection process including rationale for exclusion is presented in a flow diagram in Figure 1. SDI scores varied by SHARE country ranging from as low as 0.75 to as high as 0.91, which are all in the “High SDI” global quintile. The SDI values were dichotomised into scores below the medium value (<0.8204) and scores over the median (>0.8204) to facilitate analysis. SDI scores are presented graphically by country in Figure 2. In all, 16,657 participants from eight countries scored above the median (Austria, Belgium, Denmark, France, Germany, Netherlands, Sweden, and Switzerland) and 8073 scored below the median (Czechia, Italy, Poland, and Spain). The characteristics of the sample overall and compared by country SDI value are presented in Table 1. The mean age of the total sample included was 65.65 years (standard deviation = 10.27), and most (54%) were female and had received post-primary school education (68%). There were statistically significantly lower levels of education in those participants living in countries with high–middle SDI versus high SDI scores, with just 48% attaining post-primary level versus 82% (*p* < 0.001). Mean CASP-12 scores were 36.52, reflecting generally high QoL, albeit this was lower in those participants resident in countries with high–middle SDI scores versus high values (mean 34.32 versus 38.17, respectively; *p* < 0.001). Reflecting this, those in the high–middle countries were statistically significantly more likely to report lower SRH (49% versus 35%, respectively; *p* < 0.001).

### 3.1. Association Between SDI and Frailty

**Figure 3:**
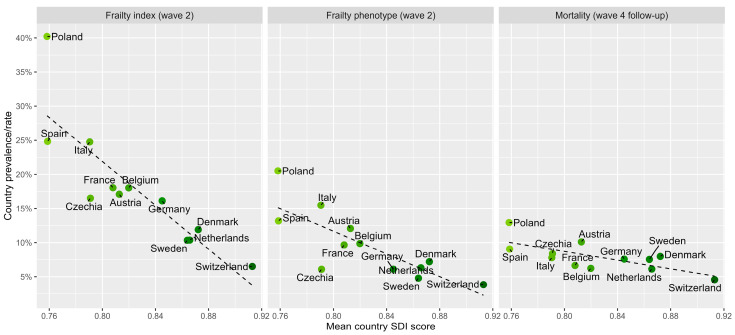
Unadjusted linear association between country Sociodemographic Index (SDI) and frailty prevalence (frailty index and frailty phenotype) as well as Wave 4 mortality follow-up (approximately 4 years later).

Frailty prevalence varied by definition and by country. In all, 21% were frail using the FI-70 while only 11% were frail based on the FP. Frailty prevalence across countries ranged from 7% to 40% using the FI-70 and from 4% to 21% using the FP. Prevalence was lowest in Switzerland and highest in Poland and showed a strong negative correlation with national SDI scores for the FI (r = −0.86) and FP (r = −0.80). There was a generally linear correlation between SDI values and frailty prevalence for countries for both the FP and FI models of frailty; see Figure 3. Those living in countries with higher SDI values (above the median value of 0.82) had statistically significantly lower frailty levels according to both measures: 16% versus 28% for those in countries with an SDI below the median (*p* < 0.001) on the FI-70, and 8% compared to 15%, respectively (*p* < 0.001), applying the FP. After adjustment for age and sex, higher SDI remained independently associated with lower odds of frailty using both the FI-70 (OR = 3.59; 95% CI: 2.96–4.34; *p* < 0.001) and the frailty phenotype (OR = 3.65; 95% CI: 2.84–4.69; *p* < 0.001). These ORs are presented in Table 2.

### 3.2. Association Between SDI and Mortality

**Figure 4:**
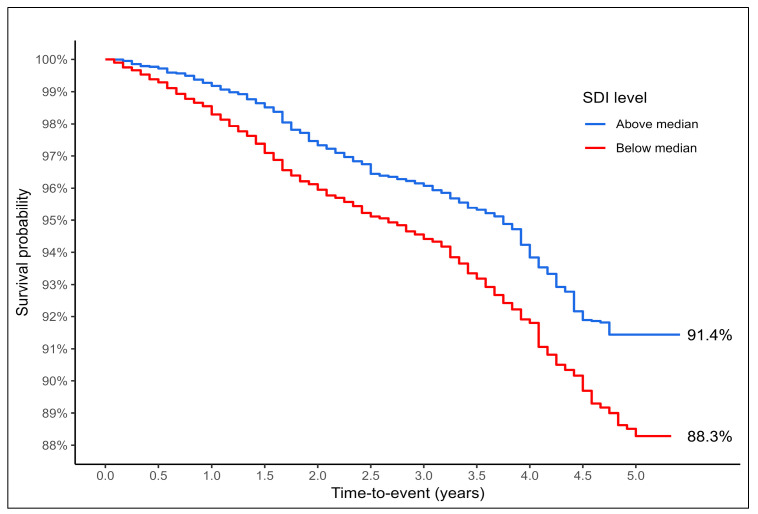
Weighted Kaplan–Meier survival curves by Sociodemographic Index (SDI) group (based on the median).

**Figure A1:**
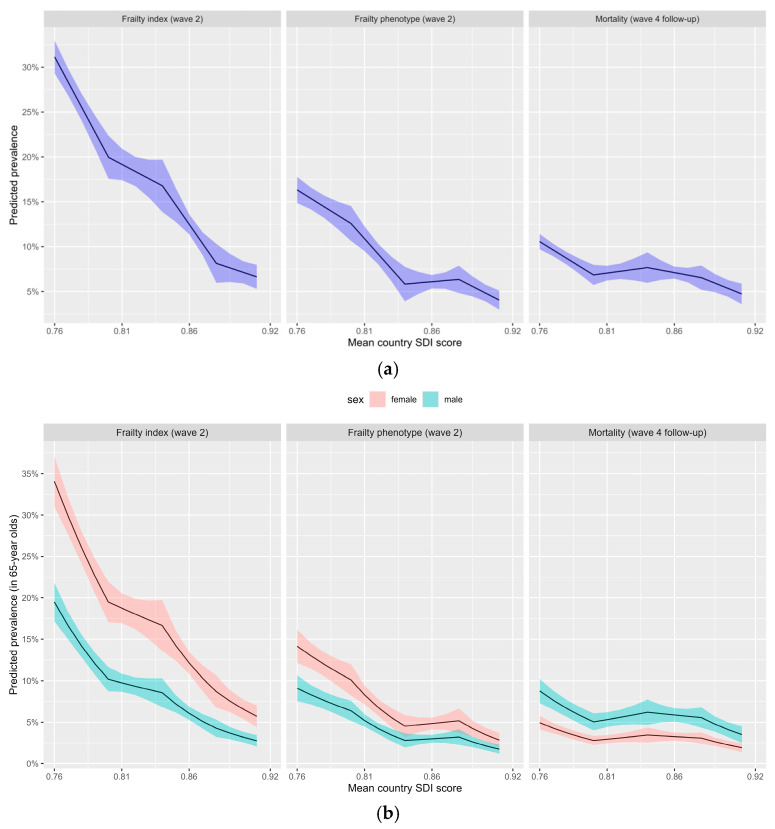
Predicted probabilities showing the association between Sociodemographic Index (SDI) score and adverse variables (frailty prevalence and mortality at Wave 4) including: (**a**) an unadjusted model including 95% confidence intervals and (**b**) an age-/sex-adjusted model with estimates presented for 65-year-old males/females. Non-linear spline model used for SDI score (three internal knots).

The mortality rate varied by countries from as low as 5% in Switzerland to 13% in Poland. Mortality was lower in countries with an SDI below the median (7% vs. 9%), and a negative correlation was found between country-level SDI scores and mortality rate (Pearson’s correlation = −0.71). The OR for the association with mortality was statically significant, OR = 1.54 (1.30–1.84; *p* < 0.001), per 0.1 decrease in SDI scores. This remained statistically significant after adjustment for age and biological sex, OR = 1.55 (1.27–1.88; *p* < 0.001), but not by frailty status, based on the FI-70, OR = 1.11 (0.93–1.33; *p* = 0.257), or the FP, OR = 1.15 (0.96–1.38; *p* = 0.127). Examining survival, the unadjusted HR was 1.48 (1.24–1.76; *p* < 0.001) per 0.1 decrease in SDI values. The associated Kaplan–Meier curves are presented in Figure 4. This remained statistically significant when adjusted for age and sex, HR = 1.46 (1.23–1.74; *p* < 0.001), and by frailty model, providing a HR = 1.22 (1.02–1.45; *p* = 0.030), when adjusted for age, sex and by the FI-70, and a HR = 1.25 (1.05–1.49; *p* = 0.012), when adjusted for age, sex and by the FP. Predicted probabilities indicating the association between continuous SDI scores and adverse variables including mortality are presented in Appendix A, Figure A1.

## 4. Discussion

In this large, population-based study of older adults across 12 European countries, we examined the relationship between country-level SDI, frailty prevalence, and mortality. Building on prior evidence linking social development to age-related disease burden and life expectancy, our findings demonstrate a strong and consistent association between lower SDI scores and higher frailty prevalence. Importantly, this relationship was observed using two conceptually distinct frailty measures, suggesting that macro-level social conditions influence multiple domains of biological ageing and vulnerability.

### 4.1. Social Development and Frailty in Ageing Populations

The SDI captures key dimensions of social development including education, income, and fertility, which are known to shape health trajectories across the life course. Our findings extend this conceptual framework by showing that countries with lower SDI scores experience a substantially higher burden of frailty among adults aged 50 years and older. The strong country-level correlation between SDI and frailty prevalence (r ≥ 0.8) suggests that frailty may represent a population-level manifestation of cumulative social disadvantage. The observed gradient in frailty prevalence across SDI categories aligns with life-course models of ageing, in which early-life and mid-life exposures, such as educational opportunities, working conditions, income security, and access to healthcare, accumulate to influence physiological reserve in later life. Countries in Europe with relatively lower SDI values (high–middle) may therefore face a dual challenge of higher frailty prevalence and fewer resources to address the complex health and social care needs of older adults. From a public health perspective, this highlights frailty as a sensitive indicator of social inequality in ageing societies and the need to develop region-specific tailored intervention strategies [26]. A key strength of this study is the use of both an FI and the FP, which reflect different conceptualizations of frailty. Despite differences in prevalence estimates based on these frailty classifications, both measures demonstrated a consistent association with SDI. This suggests that social development influences not only physical frailty but also broader dimensions of health, including comorbidity burden, functional limitations, and psychosocial wellbeing [7]. The robustness of these findings across frailty models reinforces the validity of frailty as a meaningful outcome for cross-national comparisons and public health surveillance.

### 4.2. SDI, Frailty, and Mortality

In line with the existing literature, we observed higher mortality rates in countries with lower SDI scores. However, this association was substantially attenuated after adjustment for frailty, indicating that frailty largely mediates the relationship between social development and mortality. Frailty may plausibly represent a key pathway through which social development influences survival. Lower SDI contexts are associated with cumulative exposures across the life course, including poorer education, material deprivation, and reduced access to preventive and rehabilitative care. These exposures that result in social vulnerability may accelerate the accumulation of health deficits, reduce physiological reserve, and increase susceptibility to stressors, contributing to frailty [15]. Frailty, in turn, is strongly associated with mortality through mechanisms such as impaired homeostasis, inflammation, sarcopenia, and reduced resilience to acute illness [22]. This finding supports the hypothesis that frailty may act as a critical intermediary between social context and adverse health outcomes [10]. However, while our findings support frailty as an important explanatory factor, we acknowledge that formal mediation analyses were not conducted, and therefore we interpret frailty as a potential, rather than definitive, mediating pathway. Survival analyses further demonstrated that lower SDI was associated with poorer survival independent of age and sex, but that this effect diminished once frailty was taken into account. These results underscore the central role of frailty as a proximal determinant of mortality in later life and suggest that reducing frailty prevalence may offer a pathway to narrowing survival disparities between countries with differing levels of social development [27].

### 4.3. Public Health and Policy Implications

The findings of this study have important implications for public health policy in Europe. Specifically, they demonstrate a robust association between country-level social development and frailty prevalence in Europe, irrespective of the model of frailty. Building on this evidence, and informed by a wider body of international research, policies that prioritise early identification and prevention of frailty may help mitigate the downstream consequences of social disadvantage in ageing populations. This study also found that although many European countries rank highly on global measures of social development [28], substantial heterogeneity in frailty burden, even among relatively high-SDI settings, exists [13,29]. This supports the need for continued investment in social and health systems in Europe to support healthy ageing and to prevent the accumulation of frailty and disparities across the region. For example, health promotion activities, frailty prevention and management strategies, such as promoting physical activity, improving nutrition, reducing social isolation, and optimizing chronic disease management [30,31], should be considered integral components of public health approaches to ageing across the continent [9,32,33]. Our study also identified that while mortality rates were modestly lower in higher-SDI countries, this association was attenuated after adjustment for age, sex, and frailty status, indicating that frailty itself may account for a significant proportion of observed survival differences. Indeed, monitoring frailty prevalence alongside traditional indicators such as mortality and life expectancy may provide policymakers with a more nuanced understanding of population health and ageing-related inequalities [34].

At the healthcare system level, our findings by implication support the expansion of frailty screening and early intervention strategies within primary care and community settings [31,35]. European health systems with strong primary care infrastructures may be well positioned to implement routine frailty assessment and to deliver multidisciplinary interventions that address physical, psychological, and social dimensions of vulnerability. Such approaches align with the World Health Organization’s framework on Integrated Care for Older People (ICOPE) [36] and with European strategies promoting integrated, person-centred care. At a broader level, our findings reinforce the importance of addressing upstream social determinants of health across the life course. Policies aimed at improving educational attainment, economic security, and equitable access to healthcare may have long-term benefits in reducing frailty and its associated health and social care costs with a need for public health staff to address frailty from a societal perspective rather than just taking an individual-level clinical consideration of this complex condition [37]. Finally, the cross-national variation observed in this study underscores the value of comparative European research and policy learning. Countries with lower frailty prevalence at similar levels of SDI may offer transferable lessons in health system organisation, social protection, and preventive care. Strengthening collaboration and data harmonisation across European ageing studies, such as SHARE [38], and beyond to global studies will be essential for informing evidence-based policy and for tracking progress toward healthier and more equitable ageing across the continent and worldwide [39].

### 4.4. Strengths and Limitations

This study benefits from the use of a large, harmonised, multinational dataset with standardised data collection procedures, enhancing comparability across countries. The application of two validated frailty measures strengthens confidence in the robustness of the findings. Additionally, the use of survey weights improves the generalisability of results to national populations. Nevertheless, several limitations warrant consideration. First, SDI is an aggregate, country-level indicator and cannot capture within-country socioeconomic inequalities or regional variation. As such, the observed associations may underestimate the true impact of social disadvantage at the individual level. Accordingly, caution is required when interpreting findings, as ecological bias cannot be excluded. Second, mortality ascertainment relied on proxy reports rather than linkage to official registries, which may have led to misclassification. Third, frailty was measured at a single time point, limiting our ability to assess frailty progression or recovery. Fourth, residual confounding by unmeasured factors, such as healthcare system characteristics or cultural differences in health reporting, cannot be excluded. Fifth, only a limited number of countries were included, all relatively high-income, affluent European countries, most of which are in the top 50 by SDI score globally, including the highest ranked country at the time of data collection (Switzerland), limiting the generalisability of the findings. Future research is needed to assess this by comparing countries across the world, ideally using data such as that from the GBD study for which an FI called the GBD-FI was recently developed [13,29]. Similarly, these findings are limited to country-level associations, which do not represent causation, and the reasons for these associations are difficult to determine due to the large number of differences at the country level. Sixth, large longitudinal cohort studies such as SHARE are prone to reporting and other bias. Biases may be present in the prevalence of disease and disability items which are all self-reported, and some descriptive variables had disproportional levels of missing data (such as income security and QoL), which further restricted the sample. Seventh, the exclusion of institutionalised individuals may have resulted in conservative estimates of frailty prevalence and mortality. Eighth, this analysis did not include health system-level indicators such as healthcare expenditure, primary care accessibility, or long-term care infrastructure. Omission of these factors may introduce residual confounding, potentially attenuating or inflating observed associations between SDI and frailty. Additionally, while frailty is conceptually aligned as a pathway linking social development and mortality, formal mediation analyses were not undertaken; therefore, interpretations regarding mediation should be regarded as exploratory.

## 5. Conclusions

In conclusion, this study demonstrates that lower country-level SDI scores are strongly associated with higher frailty prevalence among older adults in Europe, regardless of the frailty measure used. Although mortality rates were higher in countries with lower SDI, this relationship was largely explained by differences in frailty, highlighting frailty as a key pathway linking social development to survival. These findings emphasize the importance of incorporating frailty into public health monitoring and support the need for policies that address social determinants of health across the life course to promote healthy ageing and reduce inequalities in later life.

## Figures and Tables

**Table 1:** Descriptive statistics comparing counties included in the Survey of Health, Ageing and Retirement in Europe according to the high and high–middle Sociodemographic Index (SDI) values.

Baseline Characteristics *	Total Sample (*n* = 25,961)	Above Median (*n* = 17,313)	Below Median *(n* = 8648)	*p*-Value *
Age (years)	65.65	65.52	65.84	1
Female sex	54%	54%	55%	1
Lives alone	23%	27%	18%	<0.001
Education (post-primary) **	68%	82%	48%	<0.001
Employed **	25%	29%	20%	<0.001
Financial insecurity **	43%	29%	63%	<0.001
QoL (mean CASP-12 score) **	36.52	38.17	34.32	<0.001
Body mass index **	26.64	26.38	27.01	<0.001
Low self-rated health	41%	35%	49%	<0.001
Hospitalisation (last year)	16%	16%	15%	1
Cognitive deficit	52%	39%	69%	<0.001
Long-term health problem	51%	51%	51%	1
Disability in ADLs	20%	17%	23%	<0.001
Activities limited by health (GALI)	45%	44%	46%	0.447
Frail (frailty index)	21%	16%	28%	<0.001
Frail (frailty phenotype)	11%	8%	15%	<0.001
Mortality (at Wave 4)	8%	7%	9%	<0.001

* *p*-values adjusted for multiple comparisons (Holm method); CASP = Control, Autonomy, Self-realization, and Pleasure; GALI = Global Activity Limitation Indicator; QoL = Quality of Life. ** Note: Variables had some missing data: education (*n* = 374), employed (*n* = 6), financial insecurity (*n* = 7650), CASP-12 (*n* = 1097), and BMI (*n* = 603).

**Table 2:** Logistic regression analysis comparing SDI change (decrease) with sex and age (5-year increase) according to a 70-item frailty index, the frailty phenotype and mortality at Wave 4. The odds ratio (OR) including 95% confidence intervals (CIs).

Parameter	OR	SE	95% CI	t (8921)	*p* = x
**Frailty index**					
(Intercept)	11.83	9.09	[2.63–53.31]	3.22	0.001
SDI (0.1 decrease)	3.59	0.35	[2.96–4.34]	13.13	<0.001
Female sex	2.13	0.11	[1.92–2.36]	14.38	<0.001
Age (5-year increase)	1.56	0.02	[1.52–1.60]	33.07	<0.001
**Frailty phenotype**					
(Intercept)	3.76	3.92	[0.49–29.04]	1.27	0.204
SDI (0.1 decrease)	3.65	0.47	[2.84–4.69]	10.09	<0.001
Female sex	1.64	0.11	[1.44–1.88]	7.28	<0.001
Age (5-year increase)	1.62	0.03	[1.56–1.67]	27.05	<0.001
**Mortality (at follow-up)**					
(Intercept)	0.002	0.002	[0.00–0.01]	−7.12	<0.001
SDI (0.1 decrease)	1.55	0.15	[1.27–1.88]	4.41	<0.001
Female sex	0.54	0.04	[0.47–0.62]	−8.50	<0.001
Age (5-year increase)	1.71	0.04	[1.63–1.79]	23.57	<0.001

## Data Availability

Data may be accessed on request to the corresponding author(s).

## References

[1] GBD 2019 Ageing Collaborators (2022). Global, regional, and national burden of diseases and injuries for adults 70 years and older: Systematic analysis for the Global Burden of Disease 2019 Study. BMJ.

[2] Haagsma J.A., James S.L., Castle C.D., Dingels Z.V., Fox J.T., Hamilton E.B., Liu Z., Lucchesi L.R., Roberts N.L.S., O Sylte D. (2020). Burden of injury along the development spectrum: Associations between the Sociodemographic Index and disability-adjusted life year estimates from the Global Burden of Disease Study 2017. Inj. Prev..

[3] Hu X., Yu S.J., Gao Y.C., Zhao Y., He Y.S., Liu Y.C., Pan H.F., Wang P. (2025). Health inequality in the disease burden of non-communicable diseases among the elderly from 1990 to 2021, and projections to 2050: A systematic analysis of the Global Burden of Disease Study. BMC Geriatr..

[4] Chang A.Y., Skirbekk V.F., Tyrovolas S., Kassebaum N.J., Dieleman J.L. (2019). Measuring population ageing: An analysis of the Global Burden of Disease Study 2017. Lancet Public Health.

[5] Zheng Y., Canudas-Romo V. (2024). Global health inequality: Analyses of life disparity and healthy life disparity. Eur. J. Public Health.

[6] Wang J., Hulme C. (2021). Frailty and socioeconomic status: A systematic review. J. Public Health Res..

[7] Hanlon P., Politis M., Wightman H., Kirkpatrick S., Jones C., Khan M., Bezzina C., Mackinnon S., Rennison H., Wei L. (2024). Frailty and socioeconomic position: A systematic review of observational studies. Ageing Res. Rev..

[8] Sezgin D., O’Donovan M., Cornally N., Liew A., O’Caoimh R. (2019). Defining frailty for healthcare practice and research: A qualitative systematic review with thematic analysis. Int. J. Nurs. Stud..

[9] Hoogendijk E.O., Afilalo J., Ensrud K.E., Kowal P., Onder G., Fried L.P. (2019). Frailty: Implications for clinical practice and public health. Lancet.

[10] Andrew M.K., Dupuis-Blanchard S., Maxwell C., Giguere A., Keefe J., Rockwood K., St John P. (2018). Social and societal implications of frailty, including impact on Canadian healthcare systems. J. Frailty Aging.

[11] O’Caoimh R., Galluzzo L., Rodríguez-Laso Á., Van der Heyden J., Ranhoff A.H., Lamprini-Koula M., Ciutan M., López-Samaniego L., Carcaillon-Bentata L., Kennelly S. (2018). Prevalence of frailty at population level in European ADVANTAGE Joint Action Member States: A systematic review and meta-analysis. Ann. Ist. Super. Sanità.

[12] O’Caoimh R., Sezgin D., O’Donovan M.R., Molloy D.W., Clegg A., Rockwood K., Liew A. (2021). Prevalence of frailty in 62 countries across the world: A systematic review and meta-analysis of population-level studies. Age Ageing.

[13] O’Donovan M.R., Devleesschauwer B., Sezgin D., Liew A., Kabir Z., O’Caoimh R. (2023). Comparing frailty prevalence between countries: Validation of the Global Burden of Disease study Frailty Index (GBD-FI) in the Survey of Health, Ageing and Retirement in Europe. Age Ageing.

[14] Theou O., Brothers T.D., Rockwood M.R., Haardt D., Mitnitski A., Rockwood K. (2013). Exploring the relationship between national economic indicators and relative fitness and frailty in middle-aged and older Europeans. Age Ageing.

[15] Andrew M.K., Mitnitski A.B., Rockwood K. (2008). Social vulnerability, frailty and mortality in elderly people. PLoS ONE.

[16] Buta B.J., Walston J.D., Godino J.G., Park M., Kalyani R.R., Xue Q.L., Bandeen-Roche K., Varadhan R. (2016). Frailty assessment instruments: Systematic characterization of the uses and contexts of highly-cited instruments. Ageing Res. Rev..

[17] Fried L.P., Tangen C.M., Walston J., Newman A.B., Hirsch C., Gottdiener J., Seeman T., Tracy R., Kop W.J., Burke G. (2001). Frailty in older adults: Evidence for a phenotype. J. Gerontol. Ser. A.

[18] Mitnitski A.B., Mogilner A.J., Rockwood K. (2001). Accumulation of deficits as a proxy measure of aging. Sci. World J..

[19] Searle S.D., Mitnitski A., Gahbauer E.A., Gill T.M., Rockwood K. (2008). A standard procedure for creating a frailty index. BMC Geriatr..

[20] Cesari M., Gambassi G., van Kan G.A., Vellas B. (2014). The frailty phenotype and the frailty index: Different instruments for different purposes. Age Ageing.

[21] Börsch-Supan A., Brandt M., Hunkler C., Kneip T., Korbmacher J., Malter F., Schaan B., Stuck S., Zuber S. (2013). Data Resource Profile: The Survey of Health, Ageing and Retirement in Europe (SHARE). Int. J. Epidemiol..

[22] Theou O., Brothers T.D., Mitnitski A., Rockwood K. (2013). Operationalization of frailty using eight commonly used scales and comparison of their ability to predict all-cause mortality. J. Am. Geriatr. Soc..

[23] Olofsson J., Padyab M., Malmberg G. (2018). Health disparities in Europe’s ageing population: The role of social network. Glob. Health Action.

[24] Berger N., Van Oyen H., Cambois E., Fouweather T., Jagger C., Nusselder W., Robine J.M. (2015). Assessing the validity of the Global Activity Limitation Indicator in fourteen European countries. BMC Med. Res. Methodol..

[25] Oliver A., Sentandreu-Mañó T., Tomás J.M., Fernández I., Sancho P. (2021). Quality of life in European older adults of SHARE Wave 7: Comparing the old and the oldest-old. J. Clin. Med..

[26] Luo Y., Guo M., Zhang Q. (2025). Cross-national analysis of social determinants of frailty among middle-aged and older adults: A machine learning study in the USA, England, and China. Humanit. Soc. Sci. Commun..

[27] Dent E., Clegg A., Roller-Wirnsberger R., Vetrano D.L., Hoogendijk E.O. (2025). Reorienting frailty in clinical practice, public health, and policy: The Lancet Commission on Frailty. Lancet.

[28] Krylova P., Harmacek J., Htitich M. (2025). Social Progress Index 1990–2020: Measuring societal wellbeing over 31 years. Int. J. Soc. Res. Methodol..

[29] O’Donovan M., Sezgin D., Kabir Z., Liew A., O’Caoimh R. (2020). Assessing Global Frailty Scores: Development of a Global Burden of Disease-Frailty Index (GBD-FI). Int. J. Environ. Res. Public Health.

[30] Apóstolo J., Cooke R., Bobrowicz-Campos E., Santana S., Marcucci M., Cano A., Vollenbroek-Hutten M., Germini F., D’Avanzo B., Gwyther H. (2018). Effectiveness of interventions to prevent pre-frailty and frailty progression in older adults: A systematic review. JBI Database Syst. Rev. Implement. Rep..

[31] Travers J., Romero-Ortuno R., Bailey J., Cooney M.T. (2019). Delaying and reversing frailty: A systematic review of primary care interventions. Br. J. Gen. Pract..

[32] Cesari M., Prince M., Thiyagarajan J.A., De Carvalho I.A., Bernabei R., Chan P., Gutierrez-Robledo L.M., Michel J.-P., Morley J.E., Ong P. (2016). Frailty: An emerging public health priority. J. Am. Med. Dir. Assoc..

[33] Iburg K.M., Charalampous P., Allebeck P., Stenberg E.J., O’Caoimh R., Monasta L., Peñalvo J.L., Pereira D.M., A Wyper G.M., Niranjan V. (2023). Burden of disease among older adults in Europe—Trends in mortality and disability, 1990–2019. Eur. J. Public Health.

[34] Liotta G., Ussai S., Illario M., O’Caoimh R., Cano A., Holland C., Roller-Winsberger R., Capanna A., Grecuccio C., Ferraro M. (2018). Frailty as the future core business of public health: Report of the activities of the A3 Action Group of the European Innovation Partnership on Active and Healthy Ageing (EIP on AHA). Int. J. Environ. Res. Public Health.

[35] Galluzzo L., O’Caoimh R. (2018). Epidemiology, surveillance and population screening of frailty. Results from the systematic reviews of the European Joint Action ADVANTAGE. Preface. Ann. Ist. Super. Sanità.

[36] Won C.W., Ha E., Jeong E., Kim M., Park J., Baek J.E., Kim S., Kim S.B., Roh J., Choi J.H. (2021). World Health Organization Integrated Care for Older People (ICOPE) and the Integrated Care of Older Patients with Frailty in Primary Care (ICOOP_Frail) Study in Korea. Ann. Geriatr. Med. Res..

[37] Adja K.Y.C., Lenzi J., Sezgin D., O’Caoimh R., Morini M., Damiani G., Buja A., Fantini M.P. (2020). The importance of taking a patient-centered, community-based approach to preventing and managing frailty: A public health perspective. Front. Public Health.

[38] Pitter J.G., Zemplényi A., Babarczy B., Németh B., Kaló Z., Vokó Z. (2024). Frailty prevalence in 42 European countries by age and gender: Development of the SHARE Frailty Atlas for Europe. GeroScience.

[39] Seligman B., Ward M., Ferranna M., Bloom D.E., Kenny R.A., Orkaby A.R. (2025). A harmonized frailty index using global aging data. J. Gerontol. Ser. A.

